# The Eight Hours Day

**Published:** 1892-05-21

**Authors:** 


### PASSING TOPICS.

### THE EIGHT HOURS DAY.

Not a little serious remonstrance and still more satire have been addressed to those restless and mentally turbulent beings who are clamourous for legislation to restrict a man's output of labour to eight hours in the twenty-four. Nevertheless we suppose that it is only a question of time and importunity, and that no matter how inconsistent with sound economical conditions, or how opposed to the real interests of the community and of those who are asking for it, the fatal boon will sooner or later be yielded. And in that case it should be a point of honour with the so-called "working classes" to surrender this designation to others who may more truthfully assume it. Eight hours a day work for five days in the week, four we suppose for the sixth day, and of course no work at all on the other, with the remainder of time divided between relaxation, "culture," and sleep is a very nice programme, and when enforced would betoken the possession of leisure sufficient to elevate our working men above the actual toilers of the world.

Lord Salisbury and other notabilities have already warned the advocates of this somewhat Utopian scheme against one only too probable result of crude legislation in the direction suggested. The people who claim to pick and choose when they will work, how they will work, and what quantity of work they will undertake, may find when it is too late to repent that there is no work left for them to do. In the battle of life, as in all other battles, there are at least two parties to the strife, and simply because neither can make its dispositions safely without reference to the other, many a pretty and apparently effective manœuvre of the parade ground is abandoned as soon as the fighting becomes real. The trade of the country is only to be maintained and defended by a steadfast resolve to hold our own against all comers, and it will be taken from us as surely as we have bound ourselves to stay our hand at the stroke of the clock regardless of the state of the fray.

Yet, dropping our metaphor, it is impossible to help sympathizing with a healthy hunger for something better and more satisfying than a life brimful of toil and a sordid struggle for existence. The world would be all the better for some readjustment of the conditions of labour, and if only an equality of wisdom and a similarity of desire permitted a general concurrence, we may well believe that the daily round might be re-arranged with advantage. There is a fascination about the notion that we might gain vastly increased opportunities for recreation without sacrificing any portion of our earnings, but put plainly this only means that we would like to find that doing nothing should be paid for at the same rate as hard work, and even cosmopolitan agreement among the workers might fail to induce the paymasters of the world to acquiese in this for long together. And must not the worker deteriorate in the process? If we regard individual workers we do not find many examples of men who excel without effort. It is not how little but how much work can be done in a day that exercises a truly active mind. Innate ability and aptitude there are, but to make them of marketable value there must be nearly always diligence, and the sacrifice of mere ease. Besides, we may be sure of this, that if the programme certain leaders are now setting before the working men were honestly adhered to, and an appreciable portion of the leisure assured to them by Act of Parliament were devoted to self-culture, we should soon witness a revolt of the more intelligent against an arbitrary enactment which, under the specious pretence of providing him with time for recreation, restricted a man's liberty to make full use of the brain and muscle with which nature had endowed him.

Agricola.

